# The political empowerment of rare disease patient advocates both at EU and national level

**DOI:** 10.1186/1750-1172-7-S2-A33

**Published:** 2012-11-22

**Authors:** Terkel Andersen

**Affiliations:** 1 European Organisation for Rare Diseases (EURORDIS

## 

Thirty years ago, the patients were left with many uncertainties, no networks and no access. The rarity of the disease made it difficult to identify specialists, little information could be found. Most of the patients were engaged in a longstanding process of obtaining the right diagnosis and treatments when these were available. Significant inequality was the only standard amongst European countries.

In this context, the patients, their parents and relatives have formed clusters, reached out to other families and established patient organisations for their own diseases. The scarcity and scattered expertise on rare diseases have led patient organisations in Europe, since the early 1980s to become the link between patients and experts so to support the emergence of specific medical communities, to raise awareness and advocate for patients’ needs. Gradually, National Alliances of Rare Diseases were created to promote their rights at the national level. In March 1997, the European Organisation for Rare Diseases (EURORDIS) was established to build a strong pan-European community and critical mass to voice patients’ common needs and expectations, the European level being the most relevant to develop meaningful solutions.

People Living with Rare Diseases have shaped the concept of rare diseases as a public health policy issue: very heterogeneous diseases linked together around the specificity of rarity. Commonalities are: low prevalence, chronic, severe and often life threatening disease, limited scientific and medical knowledge base, lack of investments, winding road to diagnosis and treatment, social exclusion. Rare disease patients’ organisations are advocating across all rare diseases and all European countries. In the 1990s, new catalysing factors included scientific breakthroughs such as the mapping of the human genome and biotech therapies, and the emergence of internet as a powerful tool for information sharing.

The Committee for Orphan Medicinal Products constitutes a corner stone in developing treatments for rare diseases as much as to prompt patients’ participation in the decision making processes. Driven by a common agenda, rare disease patients promote innovative approaches to design research and healthcare policies, from medical intervention to quality of life, adapted to small populations.

Today, Rare Diseases are high on the European political agenda. European Member States are in the process of adopting national plans for rare diseases integrated within a coherent European policy framework. The active role of patients' representatives is now recognised as a major contribution to innovation and as catalyzing cooperation and sustainable development.

